# Persistent Forehead Sinus After Traditional Cautery: Frontal Mucocele Complicated by a Fronto‐Mucocutaneous Fistula

**DOI:** 10.1002/ccr3.72515

**Published:** 2026-04-12

**Authors:** Alemayehu E. Chekol, Hable D. Yigzaw, Hiwot Y. Anley, Chernet T. Mengistie, Dawit D. Yimam, Samuel Mesfin Girma

**Affiliations:** ^1^ St. Paul's Hospital Millennium Medical College‐SPHMMC Addis Ababa Ethiopia; ^2^ School of Medicine, College of Health Sciences Addis Ababa University Addis Ababa Ethiopia

**Keywords:** frontal mucocele, frontal sinus disease, mucocutaneous fistula, osteoplastic frontal sinusotomy, sinus obliteration

## Abstract

Frontal mucocutaneous fistula is a rare complication of chronic frontal sinus disease and may remain unrecognized because of its slow, insidious progression. This case report presents the clinical course, diagnostic process, and surgical management of this uncommon condition. A 56‐year‐old Ethiopian woman presented with a 5‐year history of frontal swelling, nasal obstruction, and headache. Following cautery by a traditional healer, she developed a persistent draining sinus over the right frontal region. Examination revealed a mucocutaneous fistula discharging seropurulent fluid, while laboratory studies and nasal endoscopy were unremarkable. Computed tomography (CT) demonstrated frontal sinus opacification with bony erosion consistent with a frontal mucocele complicated by fistula formation. The patient underwent external osteoplastic frontal sinusotomy with complete mucosal excision and sinus obliteration using an autologous abdominal fat graft. Postoperative recovery was uneventful, with immediate cessation of fistula drainage and complete symptom resolution. At 1‐week follow‐up, the incision had healed well with no complications. This case emphasizes the need for high clinical suspicion when evaluating chronic frontal swelling, the essential role of CT imaging in detecting sinus pathology and bony defects, and the effectiveness of surgical obliteration in achieving durable cure and preventing potentially serious intracranial sequelae.

## Key Clinical Message

A persistent, nonhealing, draining sinus on the forehead should prompt suspicion of a frontal mucocele with mucocutaneous fistula. Diagnosis relies on a high index of suspicion and CT imaging, while timely surgical obliteration is essential to prevent osteomyelitis and potentially life‐threatening intracranial complications.

## 1 Introduction

Paranasal sinus mucoceles are benign, expansile epithelial‐lined cysts that form when sinus ostia are obstructed [1, 2]. They enlarge slowly due to retained secretions, commonly affecting adults in their 30s–40s [2]. The frontal sinuses (and frontoethmoidal region) are the most frequently involved sites, accounting for roughly 35%–90% of reported cases [2, 3]. Etiologies include chronic inflammation (sinusitis, polyps), prior surgery or trauma [4]. Over time, pressure from an expanding mucocele thins and erodes the sinus walls [3]. If the mucocele becomes infected (“mucopyocele”), the expansion can accelerate and lead to complications [1, 3]. Indeed, frontal mucoceles can invade adjacent structures: orbital compression can cause proptosis and visual changes [5], and erosion into the cranial cavity may precipitate an extradural or subdural abscess [5, 6]. In the modern antibiotic era, these sequelae are uncommon, but cases still occur [1, 6].

A frontal mucocutaneous fistula (frontocutaneous fistula) is an abnormal tract between the sinus and skin, usually secondary to chronic frontal osteomyelitis [7]. This is a very rare complication of frontal sinusitis or mucocele [7]. It is akin to Pott's puffy tumor (osteomyelitis with subperiosteal abscess), which historically occurred in adolescents, though current reports are mostly in adults [6, 8]. Clinically, patients may present with a chronic forehead swelling, drainage, or sinus tract, often with minimal acute symptoms. Headache or facial pressure is common, but fever and systemic signs are often absent [3, 9]. Nasal endoscopy may reveal little if the frontal recess is blocked; thus, cross‐sectional imaging is essential. Computed tomography (CT) typically shows sinus opacification with expansile thinning and discontinuity of the frontal sinus walls [6, 10], which distinguishes it from simpler frontal cellulitis. In our patient, CT demonstrated a defect in the anterior frontal table with sinus opacification—classic for a complicated mucocele or osteomyelitis tract [6, 10].

Standard management of frontal mucocutaneous fistula involves definitive surgical drainage and sinus obliteration. Endoscopic sinus surgery (frontal sinusotomy) is often first‐line if accessible [11, 12]. However, external approaches are indicated when the disease is extensive, the frontal recess anatomy is unfavorable, or a cutaneous tract is present [6, 7]. In those cases, an osteoplastic frontal sinusotomy with fat or muscle obliteration yields excellent success [13]. We report this case to highlight the presentation and management of this rare entity in an adult.

## 2 Clinical History/ Examination

A 56‐year‐old female from rural Ethiopia presented with a 5‐year progressive frontal swelling with concurrent nasal congestion and headache. She initially sought care at a local clinic and received unspecified treatment, but no improvement occurred. When the symptoms persisted, she sought help from a traditional healer. The healer applied a hot metal instrument (“cautery”) to her forehead, claiming it would “open” the swelling. This procedure caused purulent discharge (“gush of fluid”). In the following weeks, the skin wound superficially healed, but chronic seropurulent discharge continued. This led the patient to seek hospital evaluation.

**FIGURE 1:**
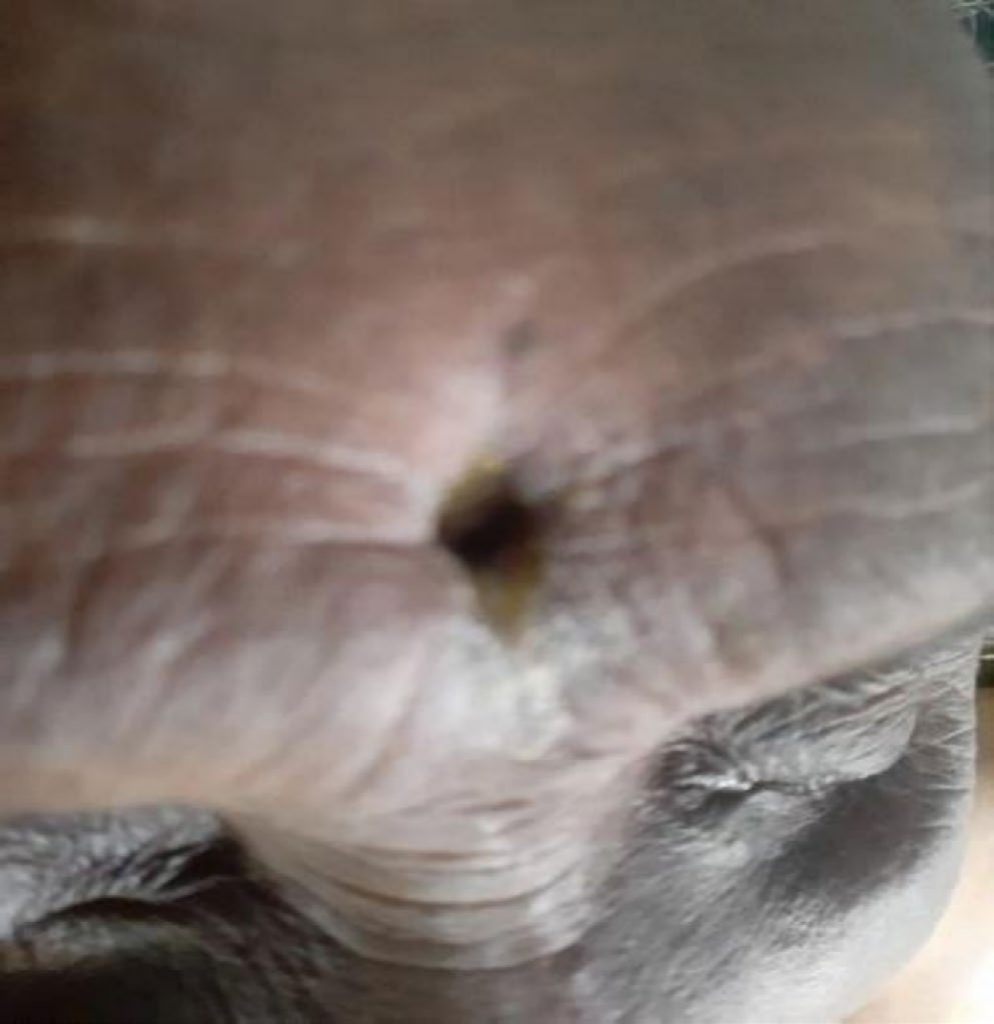
Close‐up view showing a fronto‐mucocutaneous fistula.

On examination, the patient was afebrile and had stable vital signs. On the face, over the right frontal sinus region, there was a healed circular scar with a central fistulous opening (mucocutaneous fistula) discharging thin seropurulent fluid. Surrounding skin shows no induration, fluctuating abscess, or crepitus noted (Figure 1).

## 3 Differential Diagnosis, Investigations, and Treatment

**FIGURE 2:**
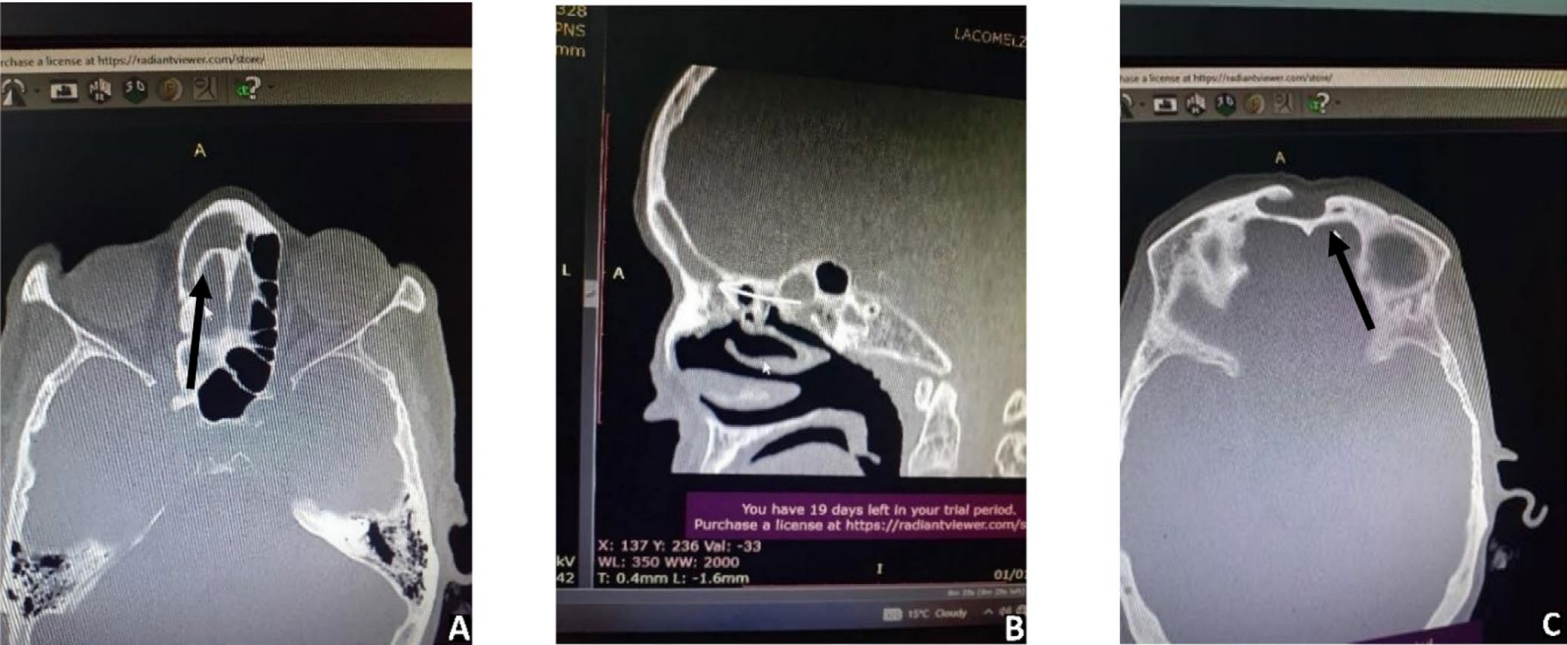
Axial CT scan of the frontal sinus showing an intact posterior table and a hypodense lesion occupying the frontal sinus cavity (A), sagittal CT image showing obstruction of the frontal sinus extending into the frontoethmoidal recess (B), axial CT scan showing a defect in the anterior table of the frontal sinus with a soft tissue density mass occupying the frontal sinus and extending into the frontoethmoidal recess (C).

Upon investigation, routine labs, including CBC, inflammatory markers, and basic metabolic panel, were within normal limits. The nasal endoscopic exam showed no abnormalities. CT scan of the head and paranasal sinuses (sagittal and axial views) revealed an anterior frontal bony plate defect with soft tissue density over the frontal sinus frontoethmoidal recess (Figure 2).

**FIGURE 3:**
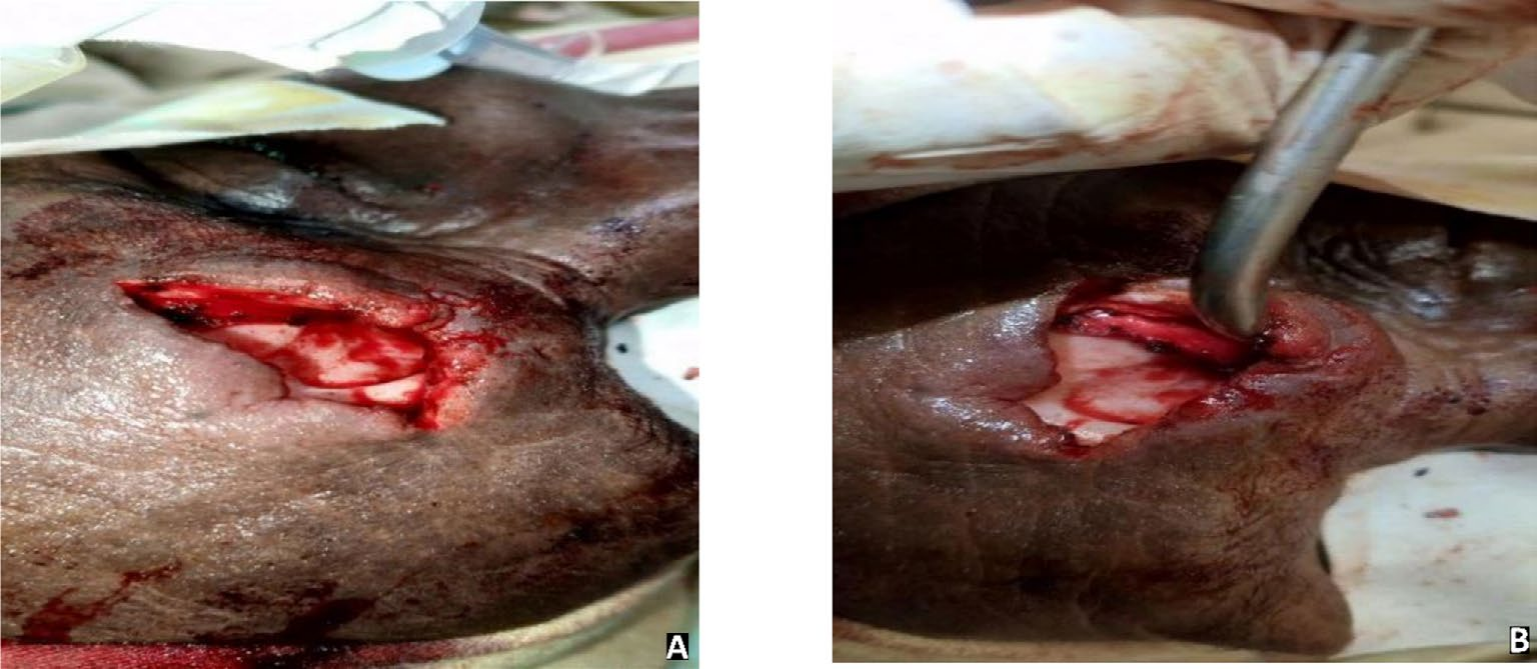
Intraoperative view of the frontocutaneous fistula during exploration from two different angles (A and B).

The patient was counseled on the need for surgical intervention. Elective open surgery (osteoplastic frontal sinusotomy with sinus obliteration) was planned due to the anterior wall defect and cutaneous tract. An external approach was employed. An elliptical skin incision was designed around the cutaneous defect, and the tract was carefully excised. The mucocutaneous junction was elevated to expose the bony margins of the frontal sinus (Figure 3). Upon entering the sinus, the frontal sinus mucosa and associated granulation tissue were completely removed. The bony walls were refreshed and curetted to ensure clearance of all diseased tissue. Both anterior and posterior tables were inspected for integrity. A fat graft, harvested from the periumbilical region, was used to obliterate the sinus cavity following meticulous hemostasis. The wound was then closed in layers with a satisfactory cosmetic outcome.

## 4 Outcome and Follow‐Up

The patient awoke from anesthesia without complications. She recovered uneventfully and was discharged on the 2nd postoperative day with outpatient follow‐up. At one‐week follow‐up, the incision was healing well, and the fistula discharge had ceased.

## 5 Discussion

Chronic frontal sinus mucoceles complicated by mucocutaneous fistula are exceedingly rare; only isolated cases and small series have been described [7, 14]. One retrospective review noted that < 1% of chronic frontal sinusitis cases developed a sinus‐to‐skin fistula [15]. Most reports come from resource‐limited settings where prolonged disease or delayed care permits osteomyelitis to develop [16]. The exact incidence is unknown due to the scarcity of published series, but the condition is clearly far less common than other sinusitis complications [14]. In practice, any chronic frontal swelling or nonhealing sinus that drains on the forehead should prompt consideration of this diagnosis [7].

Diagnostically, frontocutaneous fistulae pose a challenge because systemic or nasal findings are often subtle [14]. Patients may have a long history of mild sinus symptoms and headache [3]. In our case, the patient's chronic frontal swelling and headache led to local (nonmedical) care initially, delaying proper evaluation. At presentation, the lack of fever, normal blood work, and unremarkable nasal endoscopy can be misleading. As reported by others, inflammatory markers are frequently normal in chronic frontal sinus disease without acute infection [13]. Thus, definitive imaging is paramount: CT or MRI can identify the sinus lesion and associated bone defects [6, 11]. Our CT findings, an expansile soft tissue density in the frontal sinus with an anterior table defect, are characteristic of a chronically infected mucocele or osteomyelitis with fistulization [6, 12]. MRI (not done here) can further evaluate intracranial involvement or identify abscess components, as noted in reports [11]. In summary, a high index of suspicion and CT imaging are key to diagnosis, as this entity can be distinguished from simple abscesses or neoplasms.

Management requires surgery. Endoscopic frontal sinusotomy with wide drainage is effective in most mucoceles [11, 12]. Indeed, series reports that endoscopic marsupialization successfully treated > 89% of simple mucoceles, with recurrences in roughly 15% [12]. However, in cases like ours with anterior table erosion and a skin tract, an external approach is indicated [6, 13]. The literature supports osteoplastic frontal sinusotomy with obliteration in such scenarios: patients with posterior wall defects or osteomyelitis achieve cure only with open surgery [6]. Use of autologous abdominal fat to pack the sinus is well established; published series report > 90% long‐term success (with only 3%–10% reoperation for mucocele) [13]. In our case, fat graft obliteration after removing all mucosa and granulation tissue led to complete resolution. This is consistent with previous reports that sinus obliteration halts discharge and prevents recurrence [13, 17].

Outcomes are generally favorable when managed appropriately. Most patients experience resolution of symptoms without major complications [13, 17]. In our patient, surgery was curative: she awakened without deficits, and the fistula ceased draining within one week. Long‐term sequelae are uncommon if eradication is complete. However, delayed or inadequate treatment of frontal sinus osteomyelitis can lead to serious complications, including a frontal intracranial abscess, venous thrombosis, or meningitis [18]. In one series of epidural extension from mucoceles, urgent craniotomy was required when patients presented acutely [6]. Thus, early recognition and drainage are critical to avoid these outcomes.

## 6 Conclusion

Frontal sinus mucocutaneous fistula is an extremely rare condition resulting from chronic frontal sinus disease and osteomyelitis. Diagnosis requires vigilance: persistent forehead swelling or draining sinus should prompt imaging of the frontal sinuses. This case highlights the importance of CT in revealing sinus opacification with bony defects even when labs and endoscopy are unremarkable. Definitive treatment is surgical. Endoscopic frontal sinusotomy suffices for most mucoceles, but an open osteoplastic approach with sinus obliteration is indicated for cutaneous fistula or extensive bone erosion. When performed thoroughly, the surgical outcome is excellent, with cessation of drainage and minimal recurrence. Clinicians should be aware of this entity: prompt imaging and aggressive surgical management yield a good prognosis and prevent intracranial complications.

## Author Contributions

**Alemayehu E. Chekol:** conceptualization, writing – original draft. **Hable D. Yigzaw:** conceptualization, data curation, visualization. **Hiwot Y. Anley:** visualization, writing – review and editing. **Chernet T. Mengistie:** data curation, resources. **Dawit D. Yimam:** data curation, resources. **Samuel Mesfin Girma:** supervision, writing – review and editing.

## Funding

The authors have nothing to report.

## Ethics Statement

IRB review and approval were waived for this case report.

## Consent

Written informed consent for publication of the clinical details and accompanying images was obtained from the patient. The corresponding author holds the signed consent form and is available to the Editor upon request.

## Conflicts of Interest

The authors declare no conflicts of interest.

## Data Availability

The data supporting the findings of this study are contained within the manuscript.
